# Phase I study of pulsatile 3-day administration of afatinib (BIBW 2992) in combination with docetaxel in advanced solid tumors

**DOI:** 10.1007/s10637-012-9880-0

**Published:** 2012-11-17

**Authors:** A. H. Awada, H. Dumez, A. Hendlisz, P. Wolter, T. Besse-Hammer, M. Uttenreuther-Fischer, P. Stopfer, F. Fleischer, M. Piccart, P. Schöffski

**Affiliations:** 1 Institut Jules Bordet Brussels, Université Libre de Bruxelles, 121 Boulevard de Waterloo, Brussels, Belgium; 2 Department of General Medical Oncology and Laboratory of Experimental Oncology, University Hospitals Leuven, Leuven Cancer Institute, Catholic University Leuven, Leuven, Belgium; 3 Boehringer Ingelheim, Pharma GmbH & Co KG, Biberach, Germany

**Keywords:** Phase I, BIBW 2992, Afatinib, Epidermal growth factor receptor, Tyrosine kinase inhibitor, Pharmacokinetics

## Abstract

*Background* A phase I study to assess the maximum tolerated dose (MTD) of a short course of afatinib in combination with docetaxel for the treatment of solid tumors. *Methods* Patients with advanced solid malignancies received docetaxel 75 mg/m^2^ intravenously on day 1 and oral afatinib once daily on days 2–4, in 3-week treatment cycles. The afatinib dose was escalated in successive cohorts of 3–6 patients until dose-limiting toxicity (DLT). The MTD cohort was expanded to 13 patients. Pharmacokinetic parameters were assessed. *Results* Forty patients were treated. Afatinib doses were escalated to 160 mg/day in combination with 75 mg/m^2^ docetaxel. Three patients had drug-related DLTs during cycle 1. The MTD was defined as 90 mg/day afatinib (days 2–4) with docetaxel 75 mg/m^2^. The most frequent drug-related adverse events (all grades) were alopecia, diarrhea, stomatitis (all 50 %) and rash (40 %, all grade ≤2). Three patients had confirmed responses, two patients had unconfirmed responses and nine patients had durable stable disease >6 cycles. No pharmacokinetic interaction was observed. *Conclusion* Afatinib 90 mg administered for 3 days after docetaxel 75 mg/m^2^ is the MTD for this treatment schedule and the recommended phase II/phase III dose. This combination showed anti-tumor activity in phase I, with a manageable adverse-event profile.

## Introduction

The epidermal growth factor receptor (EGFR/ErbB) family—EGFR/human epidermal growth factor receptor (HER1/ErbB1), HER2 (ErbB2), ErbB3 (HER3) and ErbB4 (HER4)—are tyrosine kinase receptors that play an integral role in cell growth, proliferation, differentiation and migration, as well as angiogenesis through activation of complex intracellular signalling pathways [1]. Dysregulation of EGFR/HER2 expression has been observed in a variety of malignancies and is associated with more aggressive disease and poor clinical outcome [1–3].

Afatinib (BIBW 2992, Boehringer Ingelheim) is a potent orally bioavailable, irreversible ErbB-Family Blocker. Afatinib inhibits EGFR, with a half-maximal inhibitory concentration (IC_50_) of 0.5 nM, HER2 with an IC_50_ of 14 nM [4] and ErbB4 with an IC_50_ of 1 nM [5]. It has been proposed that irreversible binding to the target receptor, as well as multiple inhibition of ErbB-family members, including inhibition of trans-phosphorylation of ErbB3 [4], may help to overcome resistance that can develop with reversible small-molecule EGFR tyrosine kinase inhibitors (TKIs) or agents targeting HER2 [6]. In vivo and in vitro data suggest that afatinib is active in the L858R/T790M double mutant, which is resistant to reversible EGFR TKIs [4]. In the clinical setting, afatinib has demonstrated efficacy in patients with a range of solid tumors when administered according to different treatment schedules [7–10].

There remains an unmet need to identify improved therapeutic strategies for patients with locally advanced or metastatic tumors. One approach is the combination of TKIs with chemotherapy. Preclinical in vivo and in vitro data suggest potent anti-tumor activity of afatinib in combination with docetaxel [11]. Studies have suggested that continuous administration of TKI therapy with chemotherapy may be inferior to the administration of either agent alone [12–14]. Therefore, different scheduling strategies for the combination of these agents are needed.

Previous research has suggested that pharmacodynamic separation of EGFR TKIs and chemotherapy, specifically administration of erlotinib after chemotherapy, may lead to greater efficacy than seen with either agent alone [15–18]. In the case of erlotinib administration following docetaxel, it has been hypothesized that docetaxel induces M-phase arrest and apoptosis, which is then enhanced by erlotinib [15]. Preclinical findings support this dosing approach with afatinib. Docetaxel administration followed by afatinib was shown to inhibit tumor growth in xenograft-bearing mice more potently than afatinib followed by docetaxel [11, 19].

This phase I, dose-finding study was conducted to assess the maximum tolerated dose (MTD) and incidence of adverse events (AEs) of docetaxel (60 or 75 mg/m^2^), followed by 3 days of afatinib in a pulsatile treatment schedule.

## Materials and methods

### Study design and treatment

This phase I study was performed at two study centers in Belgium between 2005 and 2008 and was conducted in-line with the Declaration of Helsinki/International Conference on Harmonization Good Clinical Practice Guideline. It was approved by national regulatory and local ethics committees. All patients provided written informed consent.

Patients received an intravenous (IV) infusion of docetaxel on day 1 followed by three single, orally administered daily doses of afatinib on days 2–4 of each 3-week treatment cycle. Afatinib was administered in the morning (1 h prior to food intake) at a starting dose level of 10 mg and subsequently doubled until the occurrence of a drug-related dose-limiting toxicity (DLT) assessed by the Common Terminology Criteria for Adverse Events (CTCAE; version 3.0) in one or more patients. Escalation steps of a maximum of 50 % were used thereafter, unless a DLT was observed in one of six patients, in which case escalation steps were limited to ≤35 %. Docetaxel was initiated at 60 mg/m^2^ IV infusion and increased to 75 mg/m^2^ IV infusion in the absence of DLT.

A standard ‘3 + 3’ design was used. The MTD was defined as the highest dose of afatinib at which no more than one out of six patients experienced a DLT. Once the MTD was determined, a further 12 evaluable patients were included at the MTD level.

A drug-related DLT was defined as any of the following events during the first cycle: CTCAE grade 3 or 4 neutropenia of any duration associated with fever (>38.5 °C); CTCAE grade 4 neutropenia (not associated with fever for >7 days); platelets <10,000/μL or grade 3 thrombocytopenia associated with bleeding requiring transfusion; CTCAE grade 3/4 skin, central nervous system, cardiac, lung or respiratory, or hepatic AEs; grade ≥2 worsening of renal function; grade >2 diarrhea, nausea and/or vomiting for ≥7 days despite supportive care or treatment.

### Study population

Eligible patients were males or females (18 years or older) with confirmed advanced solid malignancies historically known to overexpress EGFR or HER2 who had progressed after, or were not amenable to, established treatments and for whom a therapy with proven efficacy was unavailable. Patients were required to have an Eastern Cooperative Oncology Group (ECOG) performance score of 0 or 1, life expectancy of at least 3 months, and to have recovered (CTCAE grade 0 or 1) from any drug-related AEs or previous surgery. Patients recruited at the MTD were required to have measurable disease according to the Response Evaluation Criteria in Solid Tumors (RECIST) version 1.0. Patients who had received chemo-, immune-, radio- or hormone therapy, or treatment with an EGFR/HER2-inhibiting drug within 4 weeks of study initiation, were excluded. Previous treatment with taxanes was permitted.

### Concomitant medications

Prior to taxane infusions, patients were premedicated with oral dexamethasone 8 mg twice daily for 3 days, beginning on day −1. Other concomitant treatments were permitted, as clinically necessary.

### Study assessments

Safety was evaluated by the incidence and intensity of drug-emergent AEs (defined by CTCAE, version 3.0), physical examination, changes in laboratory parameters (hematology, chemistry and urine analyses), electrocardiograms and vital signs. Objective tumor response (defined as complete [CR] or partial response [PR], stable disease [SD] or progressive disease [PD]) was assessed according to RECIST version 1.0. Target lesions were evaluated by radiography, computed tomography or magnetic resonance imaging at screening and at the end of every other cycle.

#### Pharmacokinetic sampling, data analysis and statistics

For determination of the pharmacokinetic (PK) profile of docetaxel, blood samples were collected approximately 5 min before and 1, 2, 4 and 8 h after the start of docetaxel infusion (day 1) in cycles 1 and 2. For the determination of the PK profiles of afatinib, blood samples were taken before and 1, 2, 3, 4, 5, 8 and 24 h (and 48 h only in cycle 1) after the first administration (day 2) in cycles 1 and 2. Samples collected before and 24 h after administration of afatinib were also used for docetaxel PK analysis. During cycles 3 and 4, samples were drawn before the first administration of afatinib on day 2 and 24 h after the first administration of afatinib, immediately before drug administration on day 3.

Samples were analysed using validated high-performance liquid chromatography tandem-mass spectrometry. PK parameters were calculated using WinNonlin® Professional (version 5.0.1, Pharsight® Corporation, Cary, NC, USA) and were assessed graphically and summarised by timepoint descriptive statistics using SAS.

Analyses of efficacy, safety and PK parameters are presented descriptively.

## Results

### Patient population

Forty patients received at least one dose of afatinib (treated set). Baseline characteristics are shown in Table 1. Of note, 20 % of patients had received previous taxane therapy.

**Table 1:** Patient characteristics

Characteristic	Patients
Total number of patients treated	40
Sex, *n* (%)	
Male	17 (42.5)
Female	23 (57.5)
Age, years	
Median	51.5
Range	28–79
ECOG performance status, *n* (%)	
0	9 (22.5)
1	29 (72.5)
2	1 (2.5)
3	1 (2.5)
Tumor type, *n* (%)	
Gynecologic tumors	7 (17.5)
Breast	5 (12.5)
Pancreatic	6 (15)
Skin, including melanoma	5 (12.5)
Gastrointestinal	4 (10)
Bladder	4 (10)
NSCLC	1 (2.5)
Other	8 (20)
Previous therapies, *n* (%)	
Surgery	36 (90)
Chemotherapy	39 (98)
Of which taxane therapy	8 (20)
Radiotherapy	19 (48)
Immunotherapy	3 (8)
Hormone therapy	5 (13)
Other	4 (10)
No of prior chemotherapies, *n* (%)	
0	1 (3)
1	7 (18)
2	10 (25)
≥3	22 (55)

*NSCLC* non-small cell lung cancer

### Treatment and dosing

Afatinib was studied with docetaxel 60 mg/m^2^ at 10 mg/day (*n* = 3) and with docetaxel 75 mg/m^2^ at 10 (*n* = 3), 20 (*n* = 3), 40 (*n* = 6), 60 (*n* = 4), 90 (*n* = 13), 120 (*n* = 5) and 160 (*n* = 3) mg/day. Overall, 92.5 % (*n* = 37) of patients received more than one treatment cycle, of which 11 completed eight or more treatment cycles.

### Safety and tolerability

#### Dose-limiting toxicity

DLTs during the first treatment cycle are shown in Table 2. Three patients had a DLT during the first treatment cycle. One patient experienced a DLT at 75 mg/m^2^ docetaxel and 160 mg/day afatinib—grade 4 neutropenia for 7 days that resolved without treatment. The dose of afatinib was reduced to 80 mg/day in cycle 2. Based on investigator decision, the next lower dose cohort—that is, 75 mg/m^2^ docetaxel and 120 mg/day afatinib—was expanded. Two of five patients experienced DLTs, i.e. grade 4 febrile neutropenia (one patient) and grade 3 vomiting and diarrhea (one patient). Both patients fully recovered following rescue therapy, treatment interruption or dose reduction (docetaxel 60 mg/m^2^ and afatinib 60 mg). Thereafter, another dose reduction to afatinib 90 mg and docetaxel 75 mg/m^2^ was made. When no further DLTs occurred, afatinib 90 mg was determined as the MTD and recommended phase II dose, when administered once daily for 3 days following the administration of docetaxel 75 mg/m^2^.

**Table 2:** Summary of dose-limiting toxicities during the first treatment cycle

Treatment		Dose-limiting toxicity	
Afatinib dose (mg/day)	Docetaxel dose (mg/m^2^)	CTCAE grade	Treatment cycle
120	75	Grade 4 febrile neutropenia	1
120	75	Grade 3 vomiting and diarrhea	1
160	75	Grade 4 neutropenia	1

#### Adverse events

Thirty-eight patients (95 %) experienced at least one drug-related AE following afatinib and docetaxel treatment. The most frequently reported drug-related AEs were gastrointestinal disorders in 31 patients (78 %) and skin and subcutaneous tissue disorders in 30 patients (75 %). The most frequently reported drug-related AEs (>10 % of patients) are shown in Table 3. The frequency of patients with drug-related diarrhea increased with the dose of afatinib. No grade 5 drug-related AEs occurred.

**Table 3:** Frequency of patients with drug-related AEs of grade ≥3 for related AEs occurring in >10 % of all patients

AE	CTCAE grade (*n* = 40)		
	*n* (%)		
	3	4	All grades
Alopecia	–	–	20 (50)^a^
Diarrhea	2 (5)	–	20 (50)
Stomatitis	–	–	20 (50)
Rash	–	–	16 (40)
Anorexia	2 (5)	–	11 (28)
Nausea	1 (3)	–	11 (28)
Fatigue	2 (5)	–	10 (25)
Mucosal inflammation	–	–	8 (20)
Myalgia	–	–	7 (18)
Vomiting	1 (3)	–	7 (18)
Neutropenia	2 (5)	4 (10)	6 (15)
Arthralgia	–	–	5 (13)

^a^One patient was mistakenly reported to have grade 3 alopecia

Three patients experienced drug-related AEs after treatment cycle 1 that were considered to be significant AEs. During the second treatment cycle, one patient experienced a grade 4 pulmonary embolism at 60 mg/m^2^ docetaxel and 10 mg/day afatinib, and a second patient experienced a grade 4 neutropenia at 75 mg/m^2^ docetaxel and 120 mg/day afatinib. One patient who received 75 mg/m^2^ docetaxel and 10 mg/day afatinib experienced a grade 3 infection during the seventh treatment cycle.

### Efficacy

Thirty-four of the 40 treated patients were evaluable for response according to RECIST. Fourteen patients (14/40; 35.0 %) with various tumor types had either an objective response (5/40 patients [12.5 %]; confirmed in three patients [7.5 %]) or durable (≥6 treatment cycles) SD (9/40; 22.5 %; Table 4).

**Table 4:** Summary of the characteristics of patients with objective response or durable SD (≥6 treatment cycles)

Docetaxel (mg/m^2^)	Afatinib (mg/day)	Age/gender	Tumor type	Best response	Previous taxane therapy	No. of cycles
60	10	41/F	Cervical	SD	No	10
75	10	54/F	Breast (HER2-negative)	Confirmed PR	No	7
75	20	56/M	NSCLC	SD	Yes	12
75	20	72/F	Esophagus	Confirmed PR	Yes	8
75	40	36/F	Renal	SD	No	8
75	40	65/F	Breast (HER2-positive)	Confirmed CR	No	8
75	60	52/F	Ovarian	SD	Yes	8
75	60	49/F	Breast (HER2-negative)	SD	Yes	8
75	60	59/M	Bladder	SD	No	8
75	90	46/M	Pancreas	SD	No	6
75	90	55/M	Melanoma	SD	No	14
75	90	62/F	Breast	PR	No	10
75	120	38/M	Stomach	SD	No	12
75	120	64/M	Thymus	PR	No	3

*F* female; *M* male

A CR was achieved in a patient with HER2-positive breast cancer treated with docetaxel 75 mg/m^2^/afatinib 40 mg at the end of cycle 6. Response was maintained through cycle 8. This patient had been previously treated with four cycles of doxorubicin in combination with cyclophosphamide. Two patients had a confirmed PR. One female patient with esophageal cancer treated with docetaxel 75 mg/m^2^ and afatinib 20 mg achieved a confirmed PR at the end of cycle 8. This patient had previously received seven cycles of cisplatin (two cycles)/carboplatin (five cycles) and 5-fluorouracil, and 16 courses of weekly paclitaxel. A further breast cancer patient who had previously achieved a PR to six cycles of cyclophosphamide, doxorubicin and 5-fluorouracil, achieved a confirmed PR on 75 mg/m^2^ docetaxel/10 mg afatinib. Two further patients (breast cancer and thymoma) achieved PRs that were not confirmed by a subsequent scan.

Of the eight patients that had received previous taxane therapy, three showed prolonged SD (Table 4).

### Pharmacokinetics

**Fig. 1:**
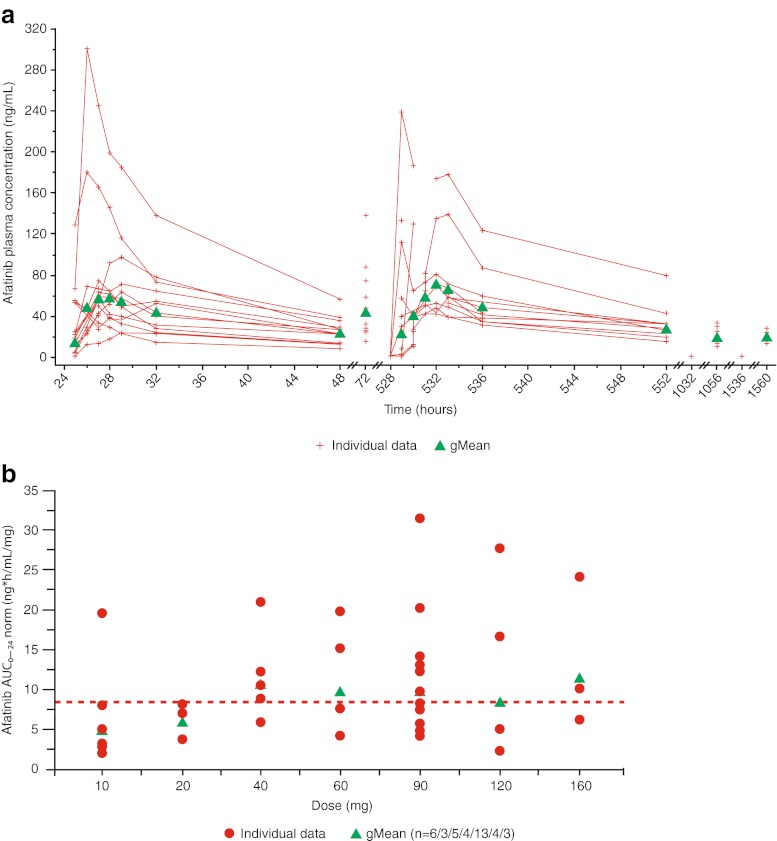
**a** Individual and gMean plasma concentration–time profiles of afatinib;* **b** Individual and gMean dose-normalized AUC_0–24_ values of afatinib.^†^ *After multiple oral administration of 90 mg afatinib tablets on days 2–4 of treatment cycles 1–4. ^†^After single oral administration of 10–160 mg afatinib tablets on day 2 of treatment cycle 1 (overall gMean: 8.44; *n* = 38). Abbreviations: gMean = geometric mean; AUC_0–24_ = area under the concentration–time curve of the analyte in plasma over the time interval from 0 to 24 h

In the MTD group, disposition kinetics of afatinib between patients in cycles 1 and 2 appeared to be comparable (Fig. 1a). Afatinib was detectable in pre-dose plasma samples in five out of 10 MTD patients on day 2 of cycle 2 after the washout phase of 18 days. Afatinib plasma concentrations increased with increasing doses in all dose groups (Fig. 1b). The PK parameters of afatinib at the MTD are shown in Table 5.

**Table 5:** Comparison of PK parameters of afatinib and docetaxel for the MTD group (75 mg/m^2^ docetaxel and afatinib 90 mg/day) by treatment cycle

Afatinib pharmacokinetics							
Parameter	Unit	Treatment cycle 1 (*n* = 13)		Treatment cycle 2 to 4 (*n* = 10)		gMean ratio; cycle 2 to cycle 1	
		gMean	gCV (%)	gMean	gCV (%)	Ratio (%)	90 % CI (%)
AUC_0–24_	ng•h/mL	879	62.6	995	48.3	122	95.5–154.7
C_max_	ng/mL	71.4	69.5	81.2	56.7	123	94.5–160.2
t_max_^a^	h	3.00	1.00–5.00	4.03	1.00–5.00	–	–
CL/F	mL/min	1040	57.5	888	52.5	–	–
V_z_/F	L	1500	70.5	1340	50.0	–	–
	
Docetaxel pharmacokinetics							
Parameter	Unit	Treatment cycle 1 (*n* = 13)		Treatment cycle 2 (*n* = 8)		gMean ratio; cycle 2 to cycle 1	
		gMean	gCV (%)	gMean	gCV (%)	Ratio (%)	90 % CI (%)
AUC_0–∞_	ng•h/mL	3160	58.1	3260	57.7	118	94.2–146.9
AUC_0–24_	ng•h/mL	2630	61.5	2680	59.4	–	–
C_max_	ng/mL	2070	63.4	2200	42.1	115	91.3–144.2
t_max_^a^	h	1.00	0.933–1.08	1.00	0.983–1.05	–	–
t_1/2_	h	20.9	26.0	21.1	33.7	–	–
CL	mL/min	712	61.0	682	65.5	–	–
V_z_	L	1290	81.3	1250	107	–	–
V_SS_	L	488	87.6	487	83.9		–

^a^Median and range

*gCV* geometric coefficient of variation; *CI* confidence interval; *C*_*max*_ maximum plasma concentration; *t*_*max*_ time to maximum plasma concentration; *CL/F* apparent clearance of the analyte in plasma following extravascular administration; *V*_*z*_ */F* apparent volume of distribution during the terminal phase following an extravascular dose; *t*_*1/2*_ half-life; *V*_*SS*_ volume of distribution at steady-state

**Fig. 2:**
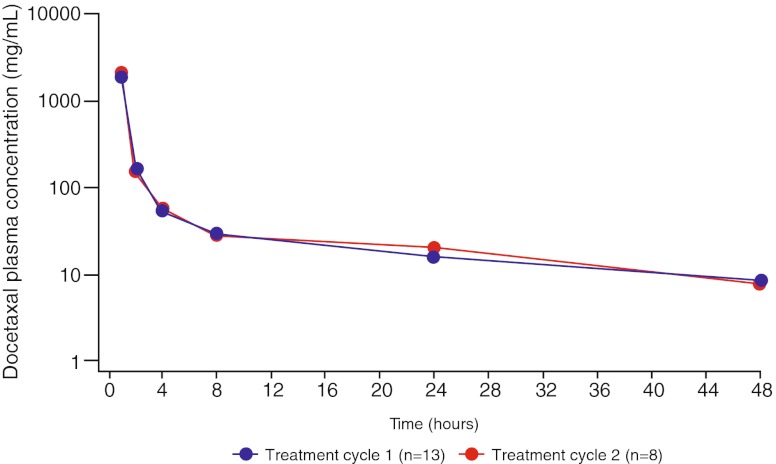
gMean plasma concentration–time profiles of docetaxel after IV administration (75 mg/m^2^) on day 1 of treatment cycles 1 and 2 in the MTD group (semi-log scale). Abbreviation: TC = treatment cycle

The gMean plasma concentrations of docetaxel and the shapes of the plasma concentration–time profiles were similar on day 1 of cycles 1 and 2 in the MTD group (Fig. 2). The PK parameters of docetaxel 75 mg/m^2^ in the MTD group are presented in Table 5. The pharmacokinetics of all patients receiving 75 mg/m^2^ docetaxel was in accordance with values for patients in the MTD group (data not shown).

## Discussion

This phase I, dose-finding study was conducted to determine the MTD and safety of 3-day pulsatile, high-dose afatinib treatment following administration of docetaxel on day 1 of a 21-day treatment cycle, a schedule selected based on preclinical data. Patients with advanced cancers historically known to overexpress EGFR and/or HER2 were preferentially eligible as they could potentially obtain greater clinical benefit from ErbB inhibition. Due to the advanced stage of the disease in many of the enrolled patients, fresh, confirmatory biopsies for EGFR and/or HER2 overexpression were not required. Results support the feasibility of this dosing schedule in patients with advanced solid tumors. Short-term, pulsatile administration of afatinib allowed for daily administration of higher afatinib doses (90 mg) than when given continuously (40 or 50 mg [9, 10, 20]). The MTD of afatinib using this regimen was significantly higher than that achieved in a previous trial evaluating the MTD of continuous afatinib (administered on days 2–21) in combination with docetaxel (administered on day 1), every 21 days. In this previous phase I study, a higher than expected incidence of hematologic side effects was observed, limiting dose escalation beyond 20 mg afatinib when given in combination with docetaxel [21].

Pulsatile administration of afatinib in combination with docetaxel was associated with manageable AEs. Diarrhea, stomatitis and alopecia were among the most common drug-related AEs reported, whereas neutropenia/febrile neutropenia were the most common DLTs. Neutropenia was also reported as a drug-related AE in 15 % of patients. Although afatinib monotherapy has not been associated with hematologic events [7–10, 20], neutropenia is a well-established DLT associated with docetaxel and rates of neutropenia reported here were comparable with those reported with docetaxel monotherapy. The observed safety profile of combined afatinib and docetaxel was consistent with documented safety observations for the individual agents [7–10, 22, 23]; no new safety concerns were identified. As might be anticipated based on cumulative exposure, the incidence and severity of diarrhea and skin rash with pulsatile administration of afatinib, as reported here, is lower than reported with continuous afatinib monotherapy [24] and, specifically, no grade 3/4 skin rash.

Pulsatile afatinib in combination with docetaxel also demonstrated promising signs of clinical activity (objective response or durable SD being observed in 35.0 % of patients). Treatment was particularly effective in patients with breast cancer and upper gastrointestinal tumors, and one patient with HER2-positive breast cancer achieved a CR. Several patients who achieved objective response or durable SD had also been previously treated with taxane therapy.

Despite inherent limitations when comparing results between studies, it is interesting to compare our findings with those of other studies combining pulsatile TKI dosing with chemotherapy. An objective response rate of 35 % has been observed with docetaxel (day 1) followed by intermittent erlotinib (days 2–16 every 3 weeks) in previously treated advanced NSCLC [15]. The most common grade 3/4 AE was neutropenia (60 %). High-dose erlotinib (days 1 and 2) with carboplatin and paclitaxel (day 3) has also been shown to have a higher response rate (34 %) compared with either low-dose erlotinib using the same schedule (18 %) or chemotherapy followed by erlotinib (28 %), again in advanced NSCLC [25]. By contrast, intermittent high-dose gefitinib given prior to docetaxel has reported much lower objective response rates (11 %) and high rates of neutropenia (61 %) [26]. Thus, work still needs to be done to fully understand the optimal dosing strategies for the co-administration of EGFR TKIs and chemotherapy.

A secondary objective of this trial was to evaluate the effect of docetaxel administration on the pharmacokinetics of afatinib and vice versa. Importantly, the afatinib exposure in the presence of docetaxel reported here appears to be comparable with that observed with afatinib monotherapy [7, 10]. Furthermore, comparison of the gMean AUC and C_max_ values of docetaxel between cycle 1 and 2 revealed only marginal differences. This is of particular importance given the complex pharmacologic profile of docetaxel and the high inter-patient variability frequently observed [27]. Moreover, the PK parameters of docetaxel reported in cycles 1 and 2 are similar to those reported in the literature [28]. Based on these data, it can be concluded that afatinib had no clinically relevant drug–drug interaction with docetaxel in the applied treatment setting. This is also in-line with data from a previous study that investigated the combination of afatinib as continuous treatment together with docetaxel [21].

In conclusion, afatinib 90 mg, when administered once daily for 3 days after administration of docetaxel 75 mg/m^2^, is the recommended dose for further clinical trials. Anti-tumor activity—that is, objective responses (12.5 %) and durable SD (22.5 %)—and a manageable side-effect profile were observed in this phase I study. No drug–drug interactions were observed between afatinib and docetaxel.
